# Supplementary material for: Encapsulation of Imazalil in HKUST-1 with Versatile Antimicrobial Activity
Source: Nanomaterials (Basel). 2022 Nov 3;12(21):3879. doi: 10.3390/nano12213879 (PMC9657569; doi:10.3390/nano12213879)
Supplement: Supplementary file 1 [file nanomaterials-12-03879-s001.zip › nanomaterials-1984339-SI.pdf]

Supporting Information

Encapsulation of Imazalil in HKUST-1 with Versatile Antimicrobial Activity

Hongqiang Dong [2,†], Yuke He [3,†], Chen Fan [4], Zhongqiang Zhu [2], Chunrong Zhang [1], Xinju Liu [1], Kun Qian [3,*] and Tao Tang [1,*]

[1] State Key Laboratory for Managing Biotic and Chemical Threats to the Quality and Safety of Agro-Products, Key Laboratory for Pesticide Residue Detection of Ministry of Agriculture and Rural Affairs, Institute of Agro-Product Safety and Nutrition, Zhejiang Academy of Agricultural Sciences, Hangzhou 310021, China
[2] College of Agriculture, Tarim University, Alaer 843300, China
[3] College of Plant Protection, Southwest University, Chongqing 400716, China
[4] Beijing Higher Institution Engineering Research Center of Food Additives and Ingredients, Beijing Technology and Business University, Beijing 100048, China
* Correspondence: qiankun1982@163.com (K.Q.); tangtao@zaas.ac.cn (T.T.); Tel./Fax: +86-571-86401292 (T.T.)
† These authors contributed equally to this work.

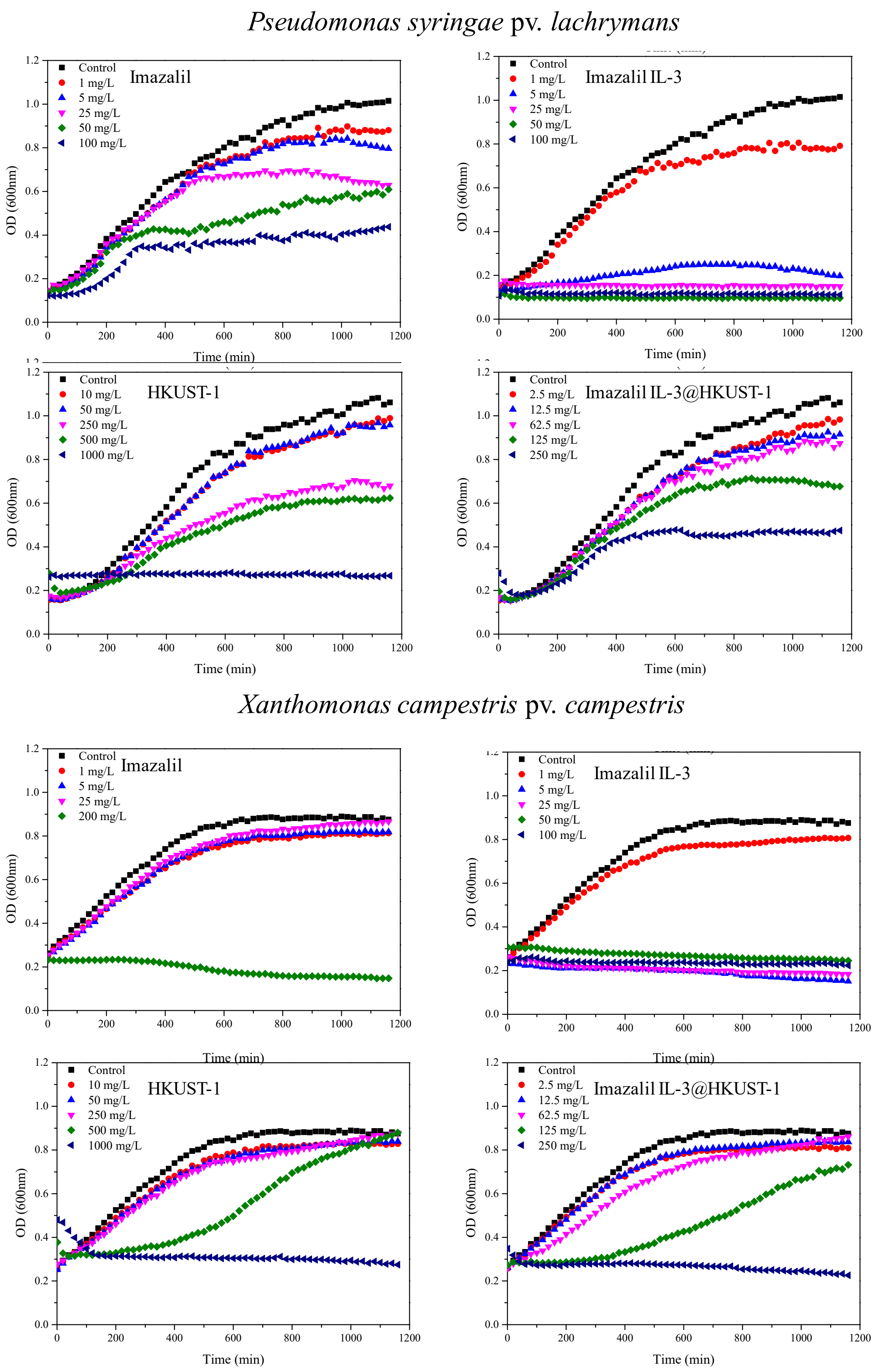

Figure S1. Antibacterial testing of imazalil, imazalil IL-3, HKUST-1 and imazalil IL-3@HKUST-1 evaluated with *Pseudomonas syringae* pv. *lachrymans* and *Xanthomonas campestris* pv. *campestris*.

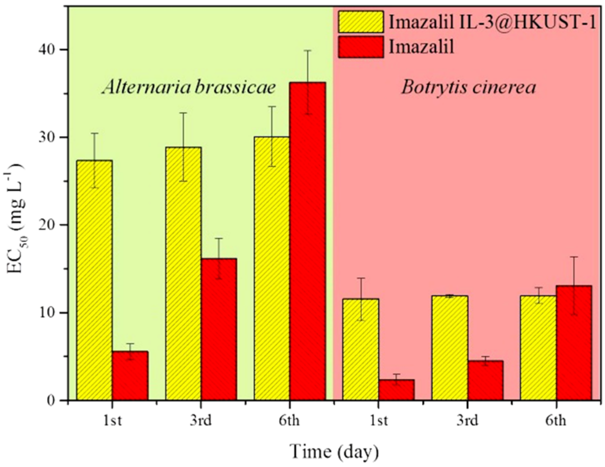

Figure S2. The activities of test compounds against *Alternaria brassicae* and *Botrytis cinerea* at different time.
